# Supplementary material for: Mixed-matrix membranes with molecular recognition windows for selective helium extraction from natural gas
Source: Nat Commun. 2026 Feb 19;17:2942. doi: 10.1038/s41467-026-69768-4 (PMC13031842; doi:10.1038/s41467-026-69768-4)
Supplement: Supplementary file 1 — Supplementary Information [file 41467_2026_69768_MOESM1_ESM.pdf]

Supplementary Information

Mixed-matrix membranes with molecular recognition windows for selective helium extraction from natural gas

Wen He,[1] Xiangzeng Wang,[2] Jian Guan,[1] Quansheng Liang,[2] Ji Ma,[1] Ying Liu,[2] Hongjun Zhang,[3] Chunwei Zhang,[2] Jiangtao Liu[1,*]

[1]CAS Key Laboratory of Urban Pollutant Conversion, Department of Environmental Science and Engineering, University of Science and Technology of China, Hefei 230026, China

[2]Shanxi Yanchang Petroleum (Group) Co., Ltd., Yanan 717599, China

[3]State Key Laboratory of Particle Detection and Electronics, University of Science and Technology of China, Hefei 230026, China

*Corresponding author

Email: jiangtaoliu@ustc.edu.cn

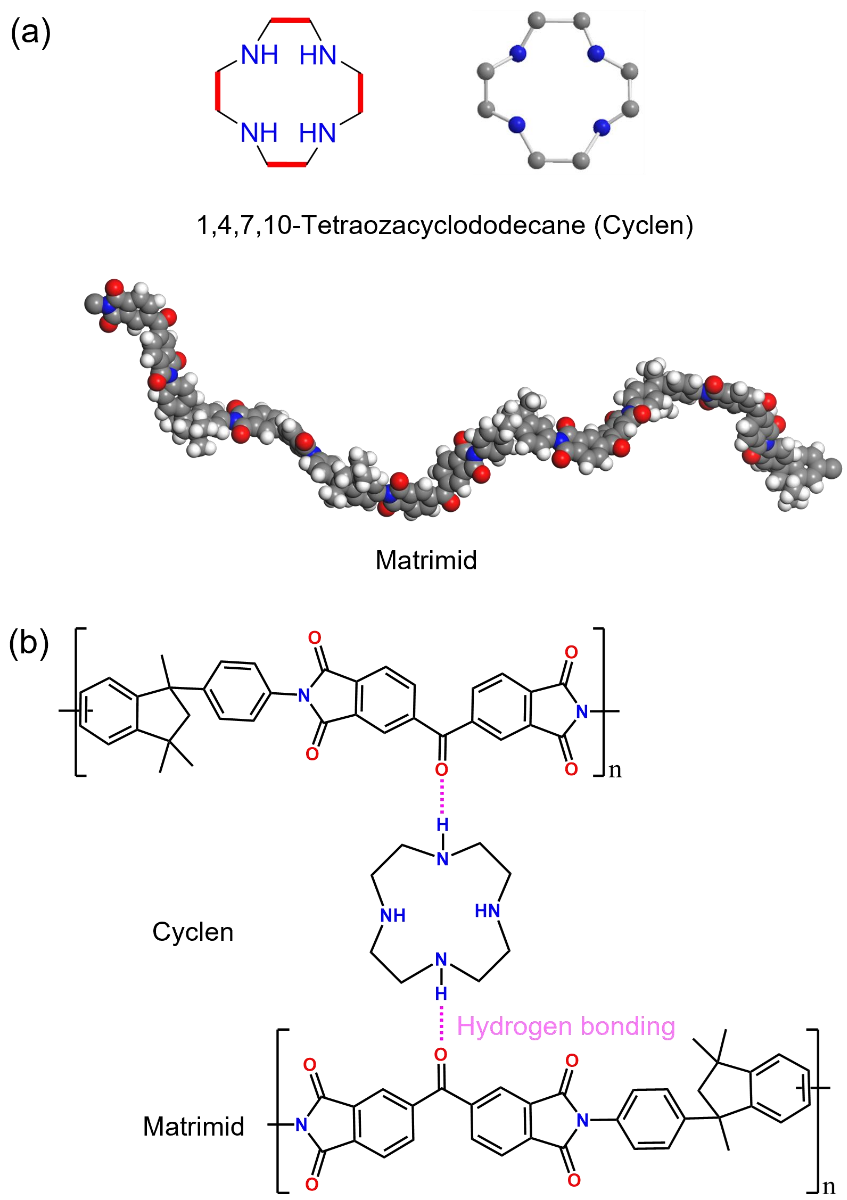

Supplementary Fig. 1 (a) Chemical structures of Matrimid and 1, 4, 7, 10-Tetraozacyclododecane (Cyclen). (b) Illustration of the interactions between Cyclen and Matrimid polymer chains.

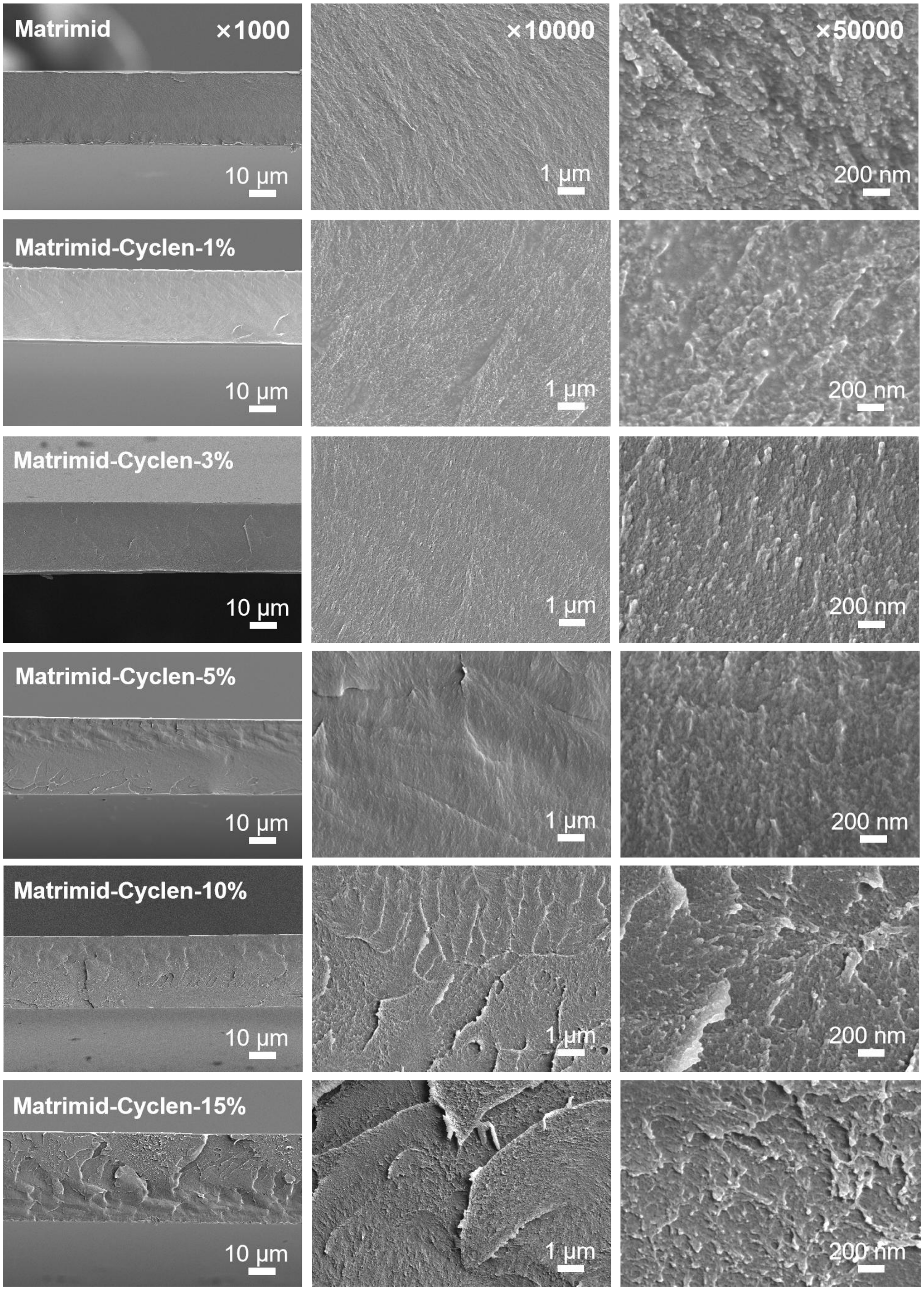

Supplementary Fig. 2 SEM images of the cross section of the Matrimid and Matrimid-Cyclen membranes up to ×50,000 magnification.

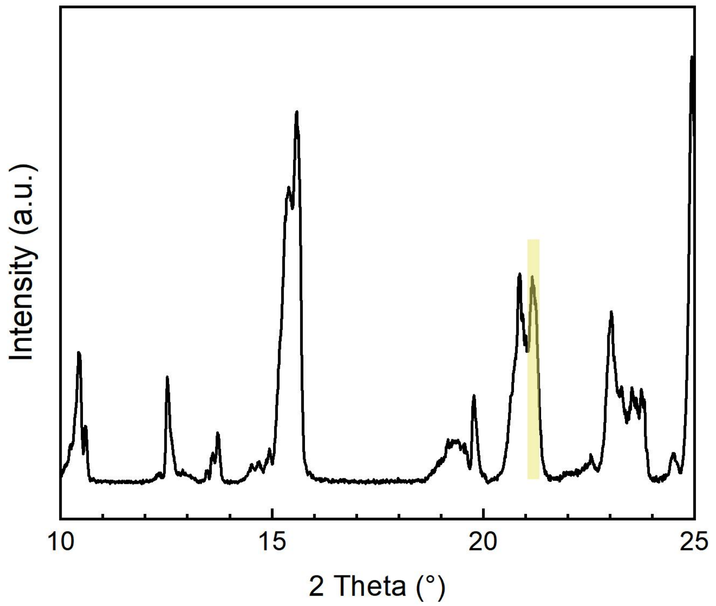

Supplementary Fig. 3 X-ray diffractometer (XRD) spectra of Cyclen powders.

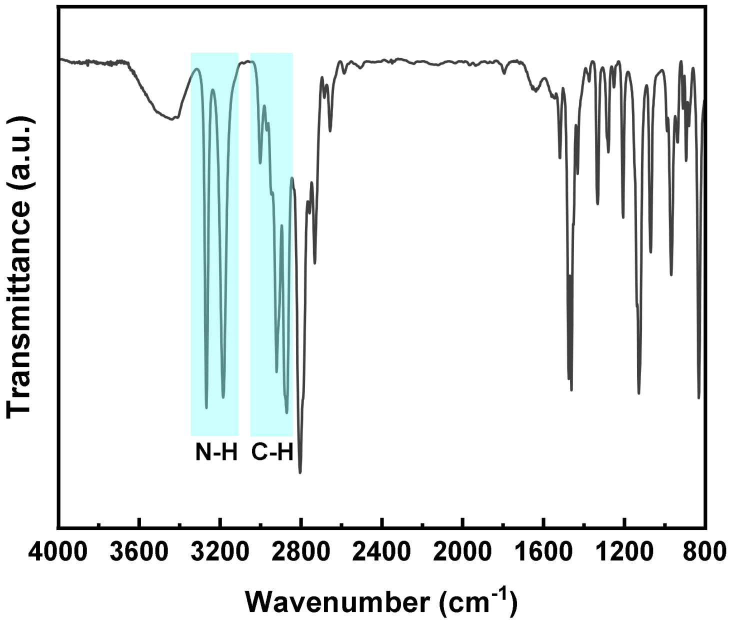

Supplementary Fig. 4 Fourier-transform infrared (FTIR) spectra of Cyclen powders.

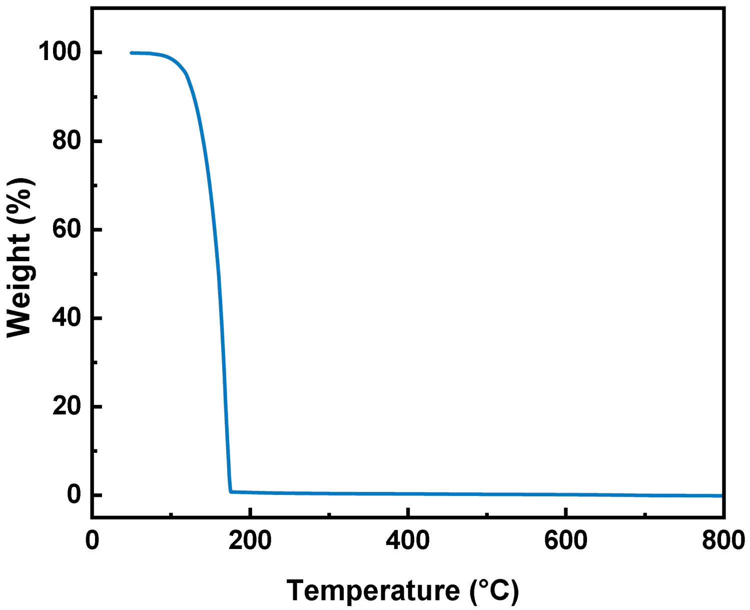

Supplementary Fig. 5 Thermogravimetric analysis (TGA) plots of Cyclen powders.

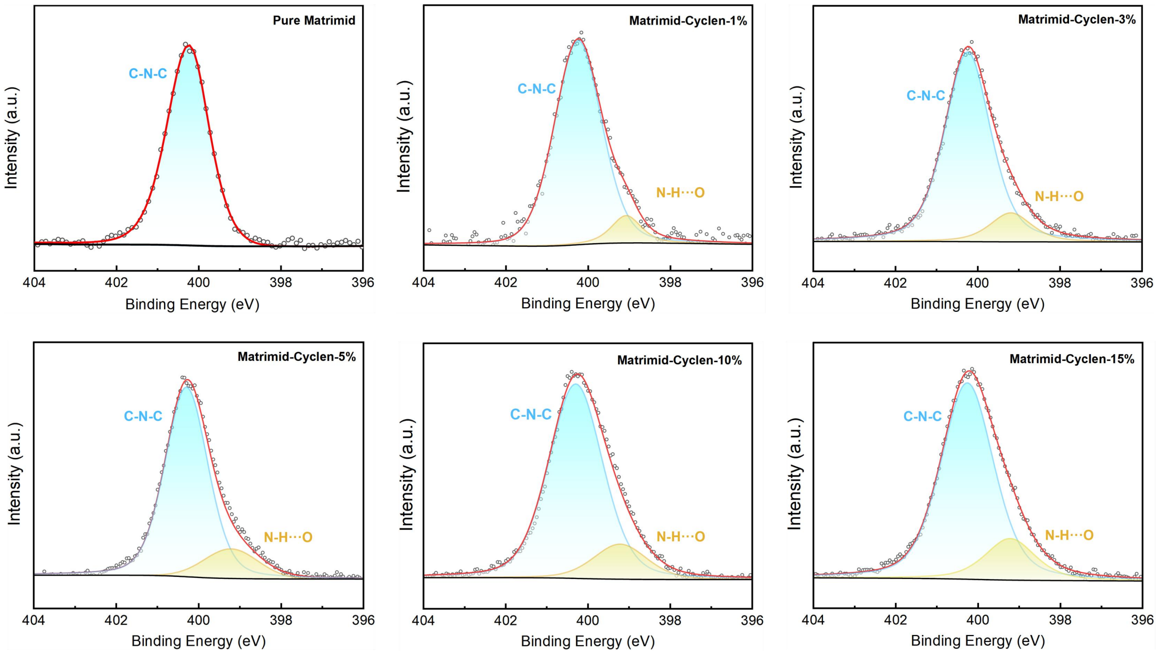

Supplementary Fig. 6 X-ray photoelectron spectroscopy (XPS) data for the N *1s* peaks of Matrimid and Matrimid-Cyclen membranes.

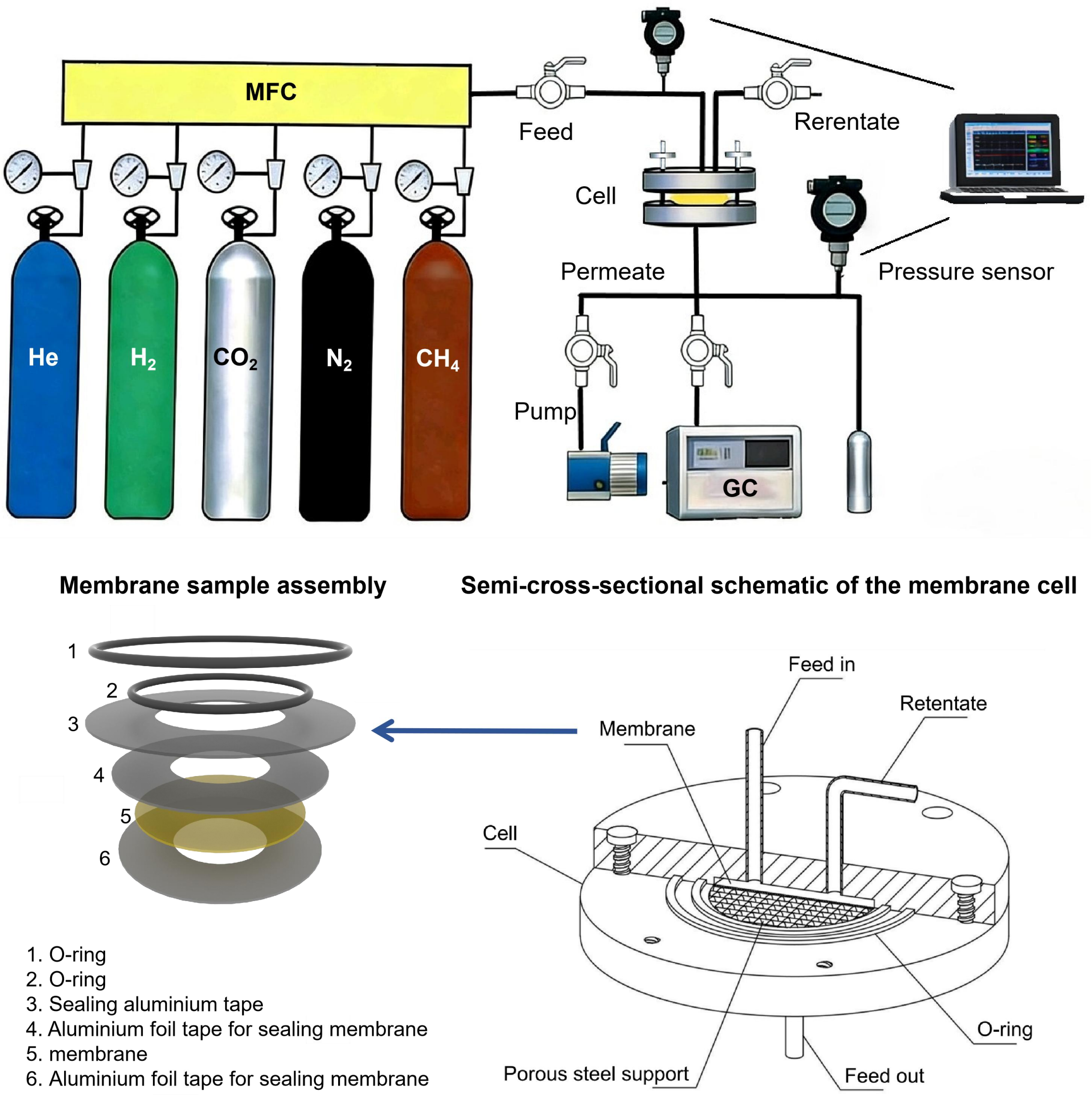

Supplementary Fig. 7 Schematic diagram of variable pressure constant-volume gas permeation cell. Abbreviations: mass flow controller (MFC), pressure regulator (PR), gas chromatography (GC).

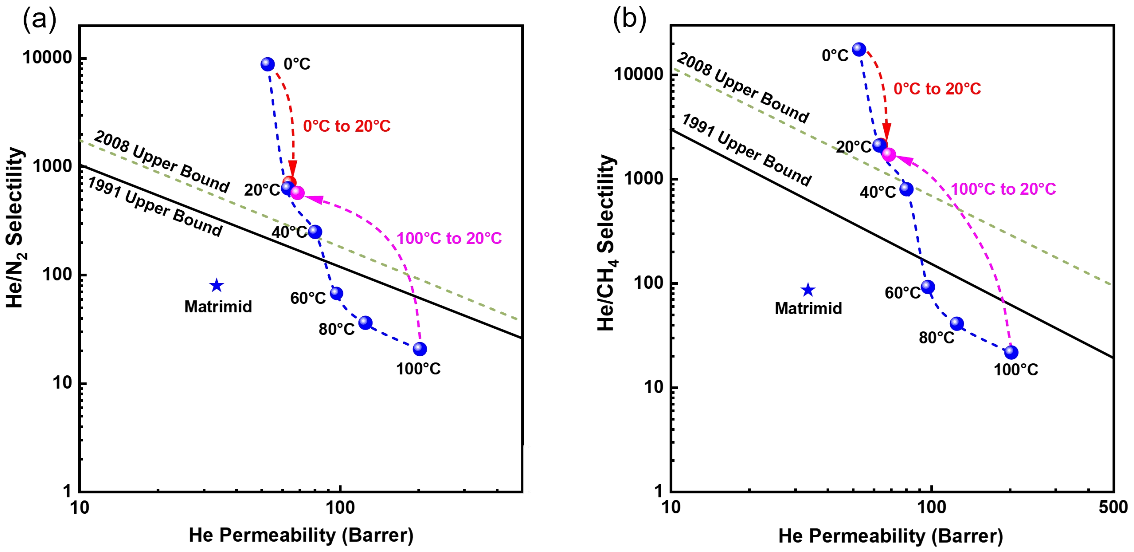

Supplementary Fig. 8 Effect of operating temperature on the separation performance of Matrimid-Cyclen-5% membrane. (a) He/N_2 and (b) He/CH_4.

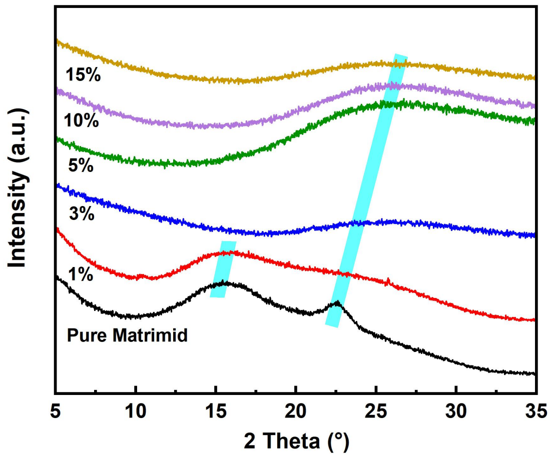

Supplementary Fig. 9 XRD patterns of the Matrimid membrane and Matrimid-Cyclen membranes after aging for 480 days.

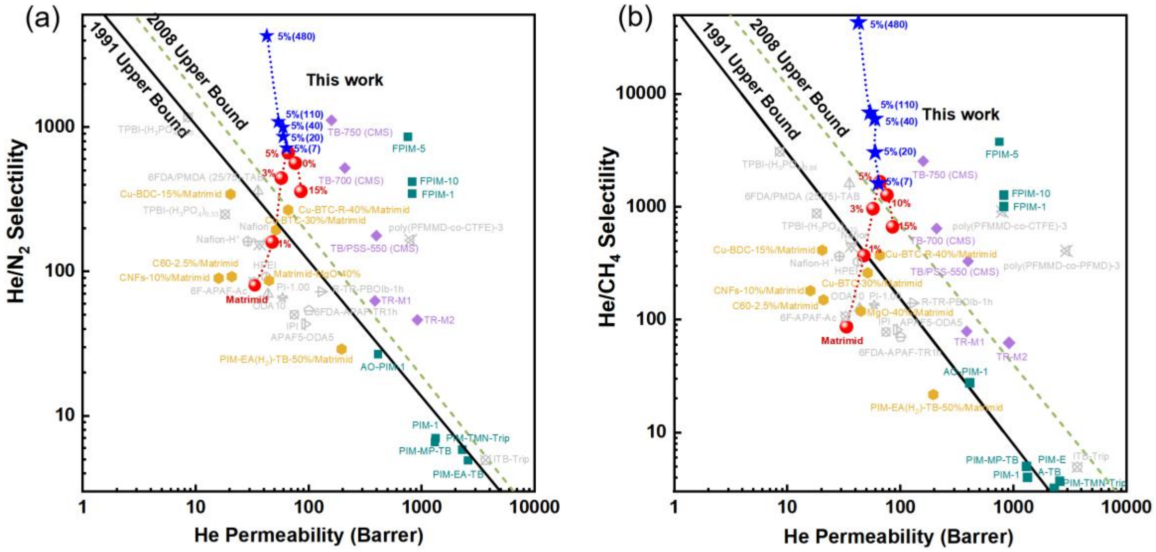

Supplementary Fig. 10 Gas separation performance of Matrimid-Cyclen membranes compared with other gas separation membranes reported for He separation. (a) He/N_2 and (b) He/CH_4. (The red symbols and blue symbols are gas data of this work, while the others are gas data of literature.)

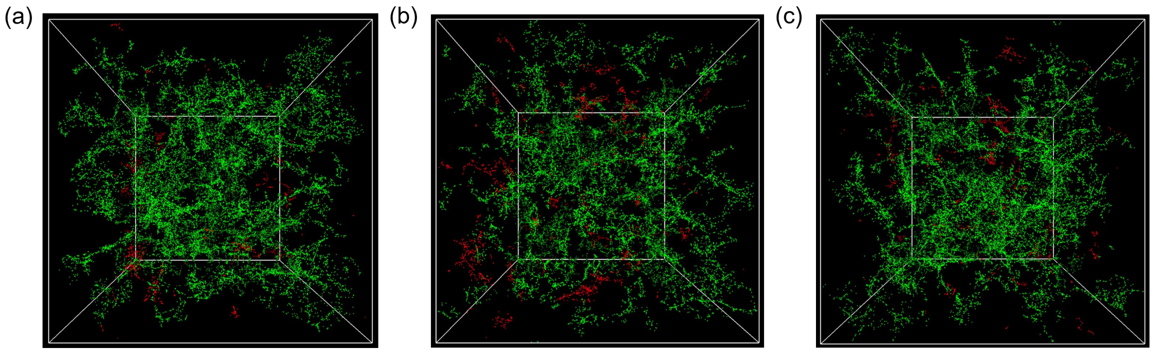

Supplementary Fig. 11 Interconnected (green) and disconnected (red) voids in Matrimid-Cyclen-n% membrane models (n% is the Cyclen mass loading, n = 0, 5, 10) with respect to a probe of 1.3 Å radius. (a) Matrimid membrane, (b) Matrimid-Cyclen-5% membrane and (c) Matrimid-Cyclen-10% membrane.

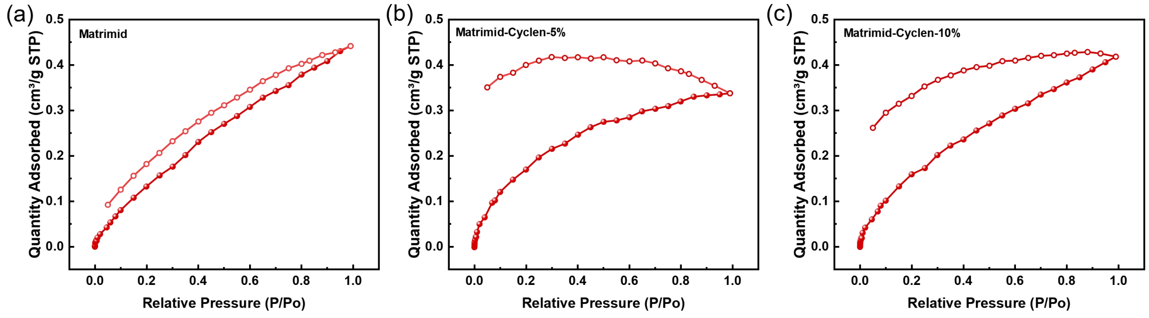

Supplementary Fig. 12 BET surface area analysis for Matrimid-Cyclen-n% membranes. H_2 sorption isotherm curves collected at 77K and corresponding pore size distribution profiles for (a) Matrimid, (b) Matrimid-Cyclen-5% and (c) Matrimid-Cyclen-10%.

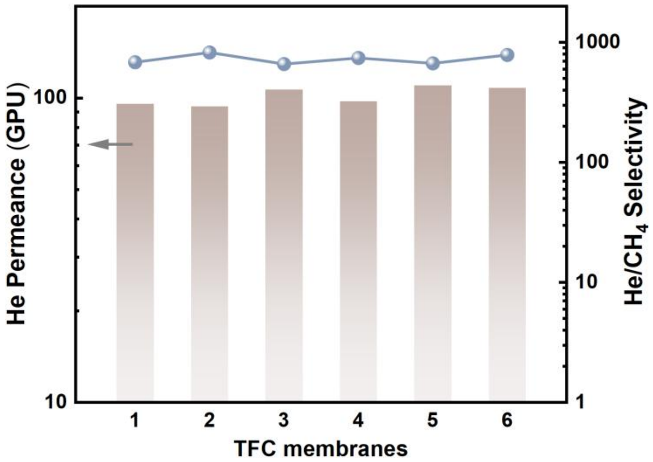

Supplementary Fig. 13 He separation performance of Matrimid-Cyclen TFC membranes (numbers 1 to 6 mean six different batches of membranes).

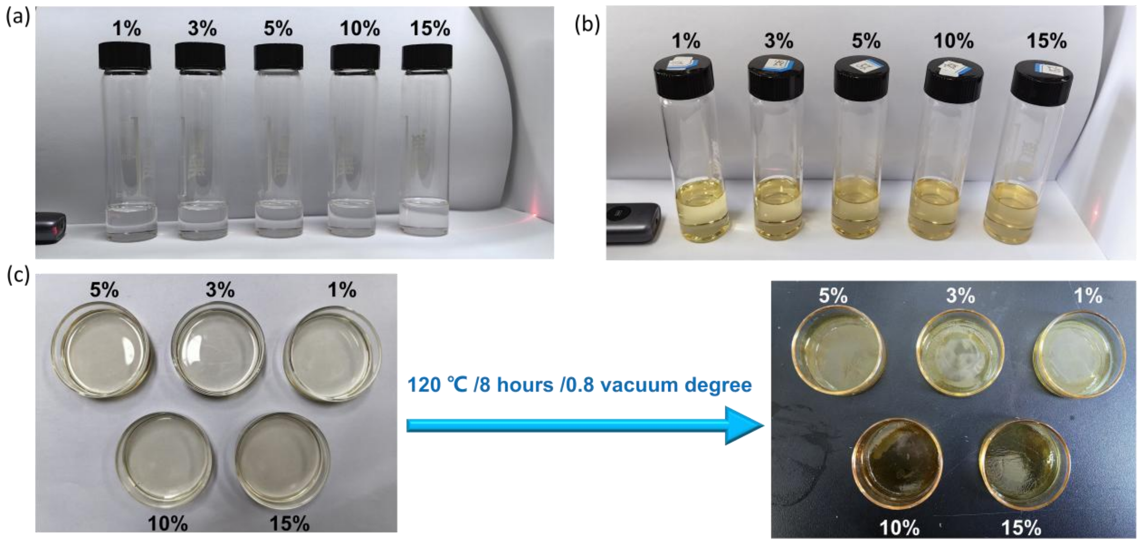

Supplementary Fig. 14 Preparation process of the Matrimid-Cyclen membranes. (a) Cyclen powder (1.0, 3.0, 5.0, 10.0 or 15.0 mg) were added to 6.0 g DMF solvent to obtain clear and transparent solutions. (b) The clean Matrimid dope solution was added to the Cyclen/DMF solution to obtain homogeneous Matrimid/Cyclen solutions. (c) The Matrimid-Cyclen solutions were injected into clean petri dishes and transferred to a vacuum oven at an ambient temperature of 120 °C for 8 hours solvent evaporation to obtain Matrimid-Cyclen membranes.

Supplementary Table 1. Normalized relative intensities (%) of nitrogen functionalities N *1s* and Oxygen functionalities O *1s* XPS spectra for Matrimid-Cyclen-n% membranes (The relevant contents were calculated from the XPS results in Fig. 3 (f) and Fig. S6).

Membrane	Nitrogen species (%)		Oxygen species (%)	
	N-C	N-H···O	C=O	C=O···H
Matrimid-Cyclen-1%	82.86%	17.14%	94.04%	5.96%
Matrimid-Cyclen-3%	78.83%	21.17%	92.33%	7.67%
Matrimid-Cyclen-5%	70.62%	29.38%	87.77%	12.23%
Matrimid-Cyclen-10%	69.77%	30.21%	85.28%	14.72%
Matrimid-Cyclen-15%	68.69%	31.31%	84.63%	15.37%

Supplementary Table 2. Summary of gas transport properties of the Matrimid and Matrimid-Cyclen-n% membranes at 1.0 atm and 25 °C (n = 1-15% indicated the degree of Cyclen mass loading).

Membrane	Gas permeability (Barrer)		
	He	H_2	CO_2
Matrimid	33.59±0.41	27.45±0.08	15.03±0.06
Matrimid-Cyclen-1%	47.87±0.24	43.04±0.16	16.90±0.06
Matrimid-Cyclen-3%	57.57±0.21	55.69±0.23	17.66±0.12
Matrimid-Cyclen-5%	66.43±0.22	62.17±0.32	20.07±0.08
Matrimid-Cyclen-10%	78.63±0.28	76.08±0.12	22.17±0.15
Matrimid-Cyclen-15%	85.93±0.80	80.85±0.30	24.76±0.07

Membrane	ideal Selectivity					
	He/N_2	He/CH_4	H_2/N_2	H_2/CH_4	CO_2/N_2	CO_2/CH_4
Matrimid	79.97±6.05	86.13±2.25	65.00±4.82	70.38±1.98	35.79±2.88	38.54±0.99
Matrimid-Cyclen-1%	145.06±10.29	368.23±28.10	143.47±9.71	331.08±26.34	56.33±3.81	130.00±10.33
Matrimid-Cyclen-3%	575.70±2.15	959.50±4.67	428.35±2.15	928.17±4.67	135.85±0.92	294.33±2.00
Matrimid-Cyclen-5%	664.30±2.10	1660.75±5.25	621.70±3.20	1554.25±8.00	200.70±0.80	501.75±3.75
Matrimid-Cyclen-10%	561.64±41.36	1310.50±4.67	543.43±40.34	1268.00±3.17	158.36±11.10	369.50±2.33
Matrimid-Cyclen-15%	358.04±12.28	661.00±48.42	336.88±11.59	621.92±49.33	103.17±3.64	190.46±14.71

Supplementary Table 3. Summary of gas transport permeability and selectivity of the Matrimid-Cyclen-5% membrane at 1.0 atm and different temperatures.

Temperature (°C)	Gas permeability (Barrer)	Selectivity	
	He	He/N_2	He/CH_4
Return to 20 °C	63.97±0.46	710.78±2.33	2132.33±7.00
0 °C	52.74±0.46	7534.29±24.28	17580.00±56.67
20 °C	63.13±0.46	631.30±2.60	2104.33±8.67
40 °C	80.14±0.46	250.44±15.13	801.40±83.82
60 °C	96.91±0.46	67.77±2.90	92.30±1.73
80 °C	125.12±0.46	36.27±0.36	41.02±0.87
100 °C	202.55±0.46	20.75±0.40	21.73±0.58
Return to 20 °C	68.69±0.27	572.42±49.85	2289.67±8.00

Supplementary Table 4. Summary of gas transport permeability and selectivity of the Matrimid-Cyclen-5% membrane at different pressures and 25℃.

Pressure (Bar)	Gas permeability (Barrer)	Selectivity	
	He	He/N_2	He/CH_4
1 Bar	66.43±0.22	664.30±2.10	1660.75±5.25
5 Bar	66.12±0.20	734.67±2.22	1652.00±6.00
10 Bar	65.48±0.13	654.80±1.30	1637.00±3.25
15 Bar	67.08±0.32	609.82±132.85	1341.60±6.40
20 Bar	68.34±0.57	525.69±39.06	976.29±8.15
25 Bar	68.28±0.34	569.00±73.14	1137.67±19.33
30 Bar	72.21±1.33	722.10±13.70	1444.20±130.02
Return to 1 Bar	66.84±0.42	668.40±4.20	1671.00±10.50

Supplementary Table 5. Summary of gas transport properties of the Matrimid and Matrimid-Cyclen-5% membrane at 1.0 atm and 25 °C during the aging process.

Membrane Aging (Days)	Gas permeability (Barrer)		
	He	H_2	CO_2
0	66.43±0.22	62.17±0.32	20.07±0.08
7	64.22±0.34	59.90±0.23	18.30±0.07
20	59.96±0.53	54.68±0.14	16.67±0.17
40	59.73±0.09	53.39±0.33	15.96±0.09
110	54.31±0.10	50.76±0.10	14.12±0.03

Membrane Aging (Days)	Selectivity					
	He/N_2	He/CH_4	H_2/N_2	H_2/CH_4	CO_2/N_2	CO_2/CH_4
0	664.30±2.10	1660.75±5.25	621.70±3.20	1554.25±8.00	200.70±0.80	501.75±3.75
7	713.56±67.960	1605.50±8.50	665.56±68.04	1497.50±21.22	203.33±21.79	457.50±1.42
20	856.57±57.74	2998.00±26.50	781.14±54.20	2734.00±6.76	238.14±18.02	833.50±9.66
40	995.50±1.50	5973.00±9.00	889.83±5.07	5339.00±30.45	266.00±1.33	1596.00±7.94
110	1086.20±2.00	6788.80±12.45	1015.20±1.74	6345.00±10.90	282.40±0.53	1765.00±3.31

Supplementary Table 6. Summary of literature He/N_2 and He/CH_4 separation performance.

Membrane Name	He Permeability (Barrer)	He/N_2 Selectivity	He/CH_4 Selectivity	Operating conditions	Gas Type	Reference
Matrimid-Cu-BTC-R-40	66.40	265.80	369.10	5.0 atm, 35 °C	Pure	1
Matrimid-Cu-BTC-30	51.80	193.40	369.10	5.0 atm, 35 °C	Pure	2
Matrimid-Cu-BDC-15%	20.50	341.70	410.00	7.0 atm, 35 °C	Pure	3
Matrimid-MgO-40%	45.00	86.1	118.4	10.0 atm, 35 °C	Pure	4
Matrimid-CNFs-10%	16.10	89.50	179.50	20.0 atm, 35 °C	Pure	5
Matrimid-PIM-EA(H2)-TB-50%	197.00	28.84	21.55	1.0 atm, 25 °C	Pure	6
Matrimid-p-xylenediamine-CL-14	21.70	112.00	155.00	10.0 atm, 35 °C	Pure	7
Matrimid-C60-2.5%	21.00	91.70	148.80	10.0 atm, 35 °C	Pure	8
HPEI	42.10	91.52	323.85	--	Pure	9
6FDA-APAF-TR1h	101.10	53.50	69.72	--	Pure	9
R-TR-PBOIb-1h	130.00	72.20	139.79	1.0 atm, 35 °C	Pure	10
APAF5-ODA5	93.20	43.14	81.04	--, 35 °C	Pure	11
ODA10	43.80	69.52	125.14	--, 35 °C	Pure	11
6F-APAF-Ac	33.00	84.00	106.00	3.0 atm, 30 °C	Pure	12
Nafion	37.00	154.17	445.78	2.0 atm, 35 °C	Pure	13
Nafion-H^+	29.10	161.67	363.75	4.0 atm, 30 °C	Pure	14
PI-1.00	59.00	65.56	134.09	2.0 atm, 35 °C	Pure	15
IPI-0.50	75.00	50.00	76.00	2.0 atm, 35 °C	Pure	15
6FDA/PMDA (25/75)-TAB	35.70	360.00	1600.00	10.0 atm, 35 °C	Pure	16
TR-M1	388.90	62.30	78.40	1.0 atm, 25 °C	Pure	17

TR-M2	921.50	46.00	62.00	1.0 atm, 25 °C	Pure	17
TB-700	210.20	519.70	639.20	2.0 atm, 25 °C	Pure	18
TB-750	160.30	1112.70	2524.40	2.0 atm, 25 °C	Pure	18
TB/PSS-550	400.20	176.60	327.30	2.0 atm, 25 °C	Pure	18
AO-PIM-1	412.00	26.80	27.60	2.0 atm, 35 °C	Pure	19
TPBI-$(H_3PO_4)_{0.33}$	18.30	248.00	870.00	6.8 atm, 25 °C	Pure	20
UiO-66-NH_2/6FAB-40%	516.00	109.30	256.70	6.8 atm, 35 °C	Pure	21
Zr-$MOP_{50wt\%}$@OPBI600	147.00	--	785.00	4.0 atm, 35 °C	Pure	22
PSU/MIL-116(Ga)-formate-20%	37.40	--	1190.00	1.0 atm, 60 °C	Pure	23
PIM-1	1336.00	7.00	4.00	--, 35 °C	Pure	24
FPIM-1	824.00	420.00	1005.00	--, 35 °C	Pure	24
FPIM-5	754.00	857.00	3770.00	--, 35 °C	Pure	24
FPIM-10	826.00	346.00	1271.00	--, 35 °C	Pure	24
PIM-EA-TB	2570.00	4.90	3.70	1.0 atm, 25 °C	Pure	25
PIM-MP-TB	1310.00	6.60	5.00	1.0 atm, 25 °C	Pure	26
PIM-TMN-Trip	2300	5.80	3.20	1.0 atm, 25 °C	Pure	27
Matrimid	33.59	79.97	86.13	1.0 atm, 25 °C	Pure	This work
Matrimid-Cyclen-1%	47.87	145.06	368.23	1.0 atm, 25 °C	Pure	This work
Matrimid-Cyclen-3%	57.57	575.70	959.50	1.0 atm, 25 °C	Pure	This work
Matrimid-Cyclen-5%	66.43	664.30	1660.75	1.0 atm, 25 °C	Pure	This work
Matrimid-Cyclen-10%	78.63	561.64	1310.50	1.0 atm, 25 °C	Pure	This work
Matrimid-Cyclen-15%	85.93	358.04	661.00	1.0 atm, 25 °C	Pure	This work

Supplementary Table 7. Summary of literature H_2/N_2 and H_2/CH_4 separation performance.

Membrane Name	H_2 Permeability (Barrer)	H_2/N_2 Selectivity	H_2/CH_4 Selectivity	Operating conditions	Gas Type	Reference
Matrimid-MOF-5	30.00	107.10	137.00	2.0 atm, 35 °C	Pure	28
Matrimid-AC	101.00	67.30	95.80	1.0 atm, 30 °C	Pure	29
Matrimid-ZSM-5	22.23	127.90	169.80	1.5 atm, 35 °C	Pure	30
Matrimid-ZIF-8	47.20	78.60	124.00	2.0 atm, 35 °C	Pure	31
Matrimid-Pd@ZIF-8	68.85	135.90	201.10	5.0 atm, 25 °C	Pure	32
Matrimid-ZIF-8-DA	430.00	45.00	42.00	3.5 atm, 35 °C	Pure	33
Matrimid-CA	34.87	102.00	117.80	2.7 atm, 35 °C	Pure	34
Matrimid-P5-SOF	150.00	75.00	70.00	1.0 atm, 20 °C	Pure	35
Matrimid-SC-3%	172.20	153.75	202.59	1.0 atm, 35 °C	Pure	36
PI-TB-4	40.00	32.10	40.00	1.0 atm, 35 °C	Pure	37
6F-AP-400	223.40	31.90	61.00	6.8 atm, 35 °C	Pure	38
6FDA-TMBDA-TBDA	339.00	21.00	34.00	--, 35 °C	Pure	39
Bio-TBPI-1	669.00	25.00	30.00	--, 35 °C	Pure	40
TBDA2-6FDA-PI	390.00	27.80	48.70	--, 35 °C	Pure	41
PIM-PI-TB-2	582.00	17.00	19.00	2.0 atm, 35 °C	Pure	42
IPD/Du-PI-0.05	604.40	19.40	26.60	3.0 atm, 35 °C	Pure	43
CoPI-TB-1	249.00	36.00	36.00	1.0 atm, 35 °C	Pure	44
PI-VUV-10	720.00	65.50	144.00	1.0 atm, 35 °C	Pure	45
5TP-FDA-Durene	861.60	17.50	22.70	6.8 atm, 30 °C	Pure	46
cross-linked ZIF-8/6FDA-durene	284.00	141.40	203.30	3.5 atm, 35 °C	Pure	47
TO-BnOCH$_3$-PI	91.90	64.70	129.40	20 atm, 35 °C	Pure	48

6FDA-1,4-trip-CF3	59.40	60.00	112.10	8.8 atm, 35 °C	Pure	49
spiroTR-PBO-BPA	19.00	42.20	35.90	1.0 atm, 35 °C	Pure	50
Psf	11.80	49.20	53.60	4.4 atm, 35 °C	Pure	51
PEI	75.80	--	16.50	1.5 atm, 25 °C	Pure	52
PPO	110.00	--	36.00	2.0 atm, 25 °C	Pure	53
Matrimid	27.45	65.00	70.38	1.0 atm, 25 °C	Pure	This work
Matrimid-Cyclen-1%	43.04	143.47	331.08	1.0 atm, 25 °C	Pure	This work
Matrimid-Cyclen-3%	55.69	428.35	928.17	1.0 atm, 25 °C	Pure	This work
Matrimid-Cyclen-5%	62.17	621.70	1554.25	1.0 atm, 25 °C	Pure	This work
Matrimid-Cyclen-10%	76.08	543.43	1268.00	1.0 atm, 25 °C	Pure	This work
Matrimid-Cyclen-15%	80.85	336.88	621.92	1.0 atm, 25 °C	Pure	This work

Supplementary Table 8. Summary of literature CO_2/N_2 and CO_2/CH_4 separation performance.

Membrane Name	CO_2 Permeability (Barrer)	CO_2/N_2 Selectivity	CO_2/CH_4 Selectivity	Operating conditions	Gas Type	Reference
Matrimid-MOF-5	20.20	40.40	44.70	2.0 atm, 35 °C	Pure	28
Matrimid-AC	66.70	23.80	29.64	1.0 atm, 30 °C	Pure	29
Matrimid-SC-3%	119.78	106.95	140.92	1.0 atm, 35 °C	Pure	36
Matrimid-POSS	5.30	53.00	37.20	10.0 atm, 35 °C	Pure	54
Matrimid-POSS+0.3M$ZnCl_2$	3.40	30.90	62.80	10.0 atm, 35 °C	Pure	54
Matrimid-ZIF-8	4.70	26.20	124.90	2.6 atm, 35 °C	Pure	55
Matrimid-CMS	12.60	33.20	51.70	3.5 atm, 35 °C	Pure	56
Matrimid-NH_2-UiO-66 ABA	37.90	--	47.70	9.0 atm, 35 °C	Mixed-50:50	57
Matrimid-TS-1	9.50	--	31.00	8.0 atm, 35 °C	Mixed-50:50	58
Matrimid-MIL-68(Al)	279.60	--	82.20	1.0 atm, 100 °C	Pure	59
Matrimid-CNT+GO	38.10	81.00	84.60	2.0 atm, 30 °C	Pure	60
Matrimid-Zeolite 13X	10.60	--	39.80	12.0 atm, 25 °C	Pure	61
Matrimid-Britesorb D300	13.30	--	26.20	12.0 atm, 25 °C	Pure	62
Matrimid-MMIF-20	10.10	--	88.00	4.0 atm, 35 °C	Mixed-50:50	62
Matrimid-MMIF-20	11.70	58.00	--	4.0 atm, 35 °C	Mixed-50:50	63
Matrimid-SAPO-34	6.90	--	67.00	10.0 atm, 25 °C	Pure	63
Matrimid-POP-2	28.00	24.00	32.00	2.0 atm, 35 °C	Pure	64
Matrimid-Cu3(BTC)2	16.00	--	61.00	5.0 atm, 35 °C	Pure	65
Matrimid-CO-3	17.10	--	102.00	2.0 atm, 35 °C	Mixed-10:90	66
Matrimid-CSM-23.3	48.60	37.60	38.00	9 atm, 35 °C	Mixed-50:50	67

Matrimid-TiO_2	10.50	11.50	13.70	2.0 atm, 35 °C	Pure	68
Matrimid-Zeolite 4A	48.30	23.40	--	2.0 atm, 25 °C	Pure	69
Matrimid-ZSM-5	423.00	86.20	--	10.0 atm, 25 °C	Pure	70
Matrimid-ZIF-8-30	28.70	17.10	24.90	4.0 atm, 22 °C	Pure	71
Matrimid-F-SPEEK	33.20	50.60	48.00	10.0 atm, 25 °C	Pure	72
Matrimid-UIO-66-NH_2	23.00	36.00	--	10.0 atm, 25 °C	Pure	73
Matrimid-PBI	305.50	19.45	52.30	3.5 atm, 35 °C	Pure	74
MOP-3/XLPEGDA50	480.00	40.00	14.00	7.8 atm, 35 °C	Pure	75
T6T6T-PEO2000	180.00	49.00	16.00	4.0 atm, 35 °C	Pure	76
Silica/XLPEG	73.10	64.10	20.90	6.0 atm, 35 °C	Pure	77
ZIF-8/PEGDA-co-PEGMEA	560.00	58.00	20.00	2.0 atm, 35 °C	Pure	78
Ethyl cellulose	67.70	21.30	11.10	2.0 atm, 25 °C	Pure	79
PPO	82.00	24.90	12.80	--, 35 °C	Pure	80
Polystyrene	14.10	28.80	18.10	4.4 atm, 23 °C	Pure	81
Polycarbonate	6.80	21.30	18.90	10 atm, 35 °C	Pure	82
Polysulfone	5.60	22.40	22.00	10.0 atm, 35 °C	Pure	83
Cellulose acetate	5.96	25.80	29.20	--, 35 °C	Pure	84
Ultem	1.48	27.40	37.00	3.5 atm, 35 °C	Pure	85
ZIF-8-PVC-g-POEM	623.00	--	11.20	1.0 atm, 35 °C	Pure	86
Cu-BTC-ODPA-TMPDA	260.70	26.10	27.76	2.0 atm, 35 °C	Pure	87
Matrimid	15.03	35.79	38.54	1.0 atm, 25 °C	Pure	This work
Matrimid-Cyclen-1%	16.90	56.33	130.00	1.0 atm, 25 °C	Pure	This work
Matrimid-Cyclen-3%	17.66	135.85	294.33	1.0 atm, 25 °C	Pure	This work

Matrimid-Cyclen-5%	20.07	200.70	501.75	1.0 atm, 25 °C	Pure	This work
Matrimid-Cyclen-10%	22.17	158.36	369.50	1.0 atm, 25 °C	Pure	This work
Matrimid-Cyclen-15%	24.76	103.17	190.46	1.0 atm, 25 °C	Pure	This work

112

113 **Supplementary Table 9.** Summary of gas transport permeability and selectivity of the

114 MMMs with different fillers.

Membrane	Gas permeability (Barrer)		Selectivity	
	He	Enhancement (%)	He/CH_4	Enhancement (%)
Matrimid-Cyclen-1%	47.87	142.51	368.23	427.53
Matrimid-Cyclododecane-1%	89.27	265.76	70.84	-17.75
Matrimid-Piperazine-1%	36.23	107.86%	157.52	182.89
Matrimid-Hexacyclen-1%	99.44	296.04	180.80	209.92

115

Supplementary Table 10. The Density, Occupied Volume and Free Volume of Matrimid-Cyclen membranes derived from simulation.

Membrane	Density (g/cm^3)	Occupied Volume ($Å^3$)	Free Volume ($Å^3$)	
			He	N_2
Matrimid	1.13	50439.11	5413.89	5141.94
Matrimid-Cyclen-5%	1.14	47949.82	6512.9	3314.1
Matrimid-Cyclen-10%	1.19	45158.94	7421.4	4324.96

Supplementary Table 11. Summary of gas transport properties for membranes of varying average thicknesses on AAO supports at 1.0 atm and 25 °C.

Membrane	Gas permeance (GPU)		
	He	H_2	CO_2
AAO supports	7747.92±115.9	6656.22±41.38	3895.34±81.29
Matrimid-Cyclen-110nm	1385.95±16.75	1111.54±25.62	458.02±45.12
Matrimid-Cyclen-1.8μm	101.93±6.41	68.32±4.16	22.58±3.11
Matrimid-Cyclen-5.6μm	18.25±0.36	15.37±0.25	5.15±0.16

Membrane	Ideal Selectivity					
	He/N_2	He/CH_4	H_2/N_2	H_2/CH_4	CO_2/N_2	CO_2/CH_4
AAO supports	1.88	2.56	1.62	2.20	0.96	1.29
Matrimid-Cyclen-110nm	58.41	66.09	46.84	53.01	19.30	21.84
Matrimid-Cyclen-1.8μm	550.95	728.04	369.30	488.00	122.05	161.29
Matrimid-Cyclen-5.6μm	829.55	1073.53	698.64	904.12	234.10	302.94

123 **Supplementary Table 12.** Summary of literature He/N_2 and He/CH_4 separation

124 performance.

Membrane Name	He permeance (GPU)	He/N_2 Selectivity	He/CH_4 Selectivity	Operating conditions	Gas Type	Reference
40nm PIM-1	23795.70	--	2.80	0.0 atm, --	Mixed-50:50	88
80nm PIM-1	21087.00	--	7.00	0.0 atm, --	Mixed-50:50	88
PIM-1	29.70	6.60	4.50	7.0 atm, 35 °C	Pure	24
PVDC-PVC	38.01	--	27.81	--, 25 °C	Pure	89
Metal oxide/PIM-PI-0	324.00	11.83	11.74	--, 21 °C	Pure	90
Metal oxide/PIM-PI-5	107.00	66.88	191.07	--, 21 °C	Pure	90
TFC-10%	24.68	--	22.34	2.0 atm, 25 °C	Pure	91
TFC-10%-450	232.10	--	47.70	2.0 atm, 25 °C	Pure	91
TFC-10%-500	916.04	--	65.24	2.0 atm, 25 °C	Pure	91
TFC-10%-550	1248.48	--	149.04	2.0 atm, 25 °C	Pure	91
TFC-2%-550	3299.49	--	33.03	2.0 atm, 25 °C	Pure	91
Ultem 1000	65.40	158.90	201.00	1.5 atm, 35 °C	Pure	92
Cellulose acetate	106.00	97.00	97.00	--, 30 °C	Pure	93
Aomatic polyamide	7.65	85.00	43.81	20.0 atm, 25 °C	Pure	94
Torlon	40.00	--	345.00	80.0 atm, 35 °C	Pure	95
P84	1.17	23.40	16.71	0.001 atm, 25 °C	Pure	96
PIM-PI-AAO-72nm	15.70	39.25	157.00	4.0 atm, 35 °C	Pure	97
PIM-PI-AAO-72nm-Age	7.70	128.33	85.56	4.0 atm, 35 °C	Pure	97
Matrimid-900	3.10	--	16700.00	7.0 atm, 35 °C	Pure	98

Matrimid-1	195.00	--	>12.00	5.0 atm, 35 °C	Pure	99
Matrimid-2	157.00	--	>109.00	5.0 atm, 35 °C	Pure	99
Matrimid-3	3313.00	--	>1.90	5.0 atm, 35 °C	Pure	99
BTPDA	643.00	--	31.40	3.0 atm, 35 °C	Pure	100
E/A-16-35℃	1577.80	--	101.80	3.0 atm, 35 °C	Pure	100
BTESE	9038.80	--	1.50	3.0 atm, 35 °C	Pure	100
$[Cu_2(bza)_4(pyz)]_n$	7.90	3.91	7.29	0.5 atm, 20 °C	Pure	101
IRMOF-3	3046.00	--	1.61	1.0 atm, --	Pure	102
MMOF	41.00	--	2.80	1.0 atm, --	Pure	103
ZIF-62	51.60	17.40	13.90	1.0 atm, 25 °C	Pure	104
SAPO-34	681.70	--	13.80	1.4 atm, --	Mixed-50:50	105
ZIF-8	565.00	4.28	4.60	1.0 atm, 24 °C	Pure	106
Zr-MOF (fumarate)	154.70	--	21.20	2.0 atm, 35 °C	Pure	107
{001 }-oriented Zr-MOF(fumarate)	1493.30	--	42.80	2.0 atm, 35 °C	Pure	107
Teflon AF 2700	10500.00	3.89	4.60	3.5 atm, 22 °C	Pure	108
Hyflon AD 60	2600.00	14.44	35.00	3.5 atm, 22 °C	Pure	108
Cytop	790.00	43.89	130.00	3.5 atm, 22 °C	Pure	108
Copolymer A	1400.00	48.28	260.00	3.5 atm, 22 °C	Pure	108
Copolymer B3	770.00	78.57	480.00	3.5 atm, 22 °C	Pure	108
Poly(PFMMD)	2160.00	31.77	108.00	3.5 atm, 22 °C	Pure	109
PFMMD-co-PFMD 2	2320.00	57.28	332.00	3.5 atm, 22 °C	Pure	109
PFMMD-co-PFMD 3	2970.00	67.50	405.00	3.5 atm, 22 °C	Pure	109
PFMMD-co-CTFE 2	1120.00	94.12	473.00	3.5 atm, 22 °C	Pure	109

PFMMD-co-CTFE 3	804.00	164.08	900.00	3.5 atm, 22 °C	Pure	109
PBDI	45	--	1380	1.0 atm, 100 °C	Mixed-50:50	110
PF-SPF	65.00	866.67	2166.67	1.0 atm, 35 °C	Pure	111
Matrimid-Cyclen-110nm	1385.95	58.41	66.09	1.0 atm, 25 °C	Pure	This work
Matrimid-Cyclen-1.8μm	101.93	550.95	728.04	1.0 atm, 25 °C	Pure	This work
Matrimid-Cyclen-5.6μm	18.25	829.55	1073.53	1.0 atm, 25 °C	Pure	This work

Supplementary Table 13. Summary of gas transport properties of the AAO supports (pore size < 20 nm), and the TFC membranes made from Matrimid and from Matrimid-Cyclen at 1.0 atm and 25 °C (Cyclen mass loading is 5%).

Membrane	Gas permeance (GPU)		
	He	H_2	CO_2
AAO supports	7747.92±115.9	6656.22±41.38	3895.34±81.29
Matrimid-1.2μm	109.50±6.94	92.22±4.75	30.90±1.52
Matrimid-Cyclen-1.8μm	101.93±6.41	68.32±4.16	22.58±3.11

Membrane	Ideal Selectivity					
	He/N_2	He/CH_4	H_2/N_2	H_2/CH_4	CO_2/N_2	CO_2/CH_4
AAO supports	1.88	2.56	1.62	2.20	0.96	1.29
Matrimid-1.2μm	44.51	55.30	37.49	46.58	12.56	15.61
Matrimid-Cyclen-1.8μm	550.95	728.04	369.30	488.00	122.05	161.29

Supplementary References

[1] A. Akbari, J. Karimi-Sabet, S.M. Ghoreishi, Intensiffcation of helium separation from CH_4 and N_2 by size-reduced Cu-BTC particles in Matrimid matrix, Separation and Puriffcation Technology, 251, 117317 (2020).

[2] A. Akbari, J. Karimi-Sabet, S.M. Ghoreishi, Matrimid® 5218 based mixed matrix membranes containing metal organic frameworks (MOFs) for helium separation, Chemical Engineering & Processing: Process Intensification, 148, 107804 (2020).

[3] A. Ali, J. Karimi-Sabet, S.M. Ghoreishi, Polyimide based mixed matrix membranes incorporating Cu-BDC nanosheets for impressive helium separation, Separation and Purification Technology, 253, 117430 (2020).

[4] S.S. Hosseini, Y. Li, T.S. Chung, Y. Liu, Enhanced gas separation performance of nanocomposite membranes using MgO nanoparticles, Journal of Membrane Science, 302 (1-2), 207-217 (2007).

[5] M. Dohade, Incorporation of carbon nanofibers into a Matrimid polymer matrix: Effects on the gas permeability and selectivity properties, Journal of Applied Polymer Science, 135 (12) 46019 (2018).

[6] E. Esposito, I. Mazzei, M.Monteleone, A. Fuoco, M. Carta, N.B. Makeown, R. Malpass-Evans, J.C. Jansen, Highly permeable Matrimid®/PIM-EA (H_2)-TB blend membrane for gas separation, Polymers, 11 (1), 46 (2018).

[7] P.S. Tin, T.S. Chung, Y. Liu, R. Wang, S.L. Liu, K.P. Pramoda, Effects of cross-linking modification on gas separation performance of Matrimid membranes, Journal of Membrane Science, 225(1-2), 77-90 (2003).

[8] T.S. Chung, S.S. Chan, R. Wang, Z.H. Lu, C.B. He, Characterization of permeability and sorption in Matrimid/C60 mixed matrix membranes, Journal of Membrane Science, 211 (1), 91-99 (2003).

[9] M. Calle, Y.M. Lee, Thermally rearranged (TR) poly (ether-benzoxazole) membranes for gas separation, Macromolecules, 44 (5), 1156-1165 (2011).

[10] Y.B. Zhuang, J.G. Seong, W.H. Lee, Y.S. Do, M,J, Lee, G. Wang, M.D. Guiver, Y.M. Lee, Mechanically tough, thermally rearranged (TR) random/block poly (benzoxazole-co-imide) gas separation membranes, Macromolecules 48 (15), 5286-5299 (2015).

[11] H.J. Jo, C.Y. Soo, G.X. Dong, Y.S. Do, H.H. Wang, M.J. Lee, J.R. Quay, M.K. Murphy, Y.M. Lee, Thermally rearranged poly (benzoxazole-co-imide) membranes with superior mechanical strength for gas separation obtained by tuning chain rigidity, Macromolecules, 48 (7), 2194-2202 (2015).

[12] B. Díez, P. Cuadrado, Á. Marcos-Fernańdez, P. Pradános, A. Tena, L. Palacio, Á.E. Lozano, A. Hernańdez, Helium recovery by membrane gas separation using poly (o-acyloxyamide) s, Industrial & Engineering Chemistry Research, 53 (32), 12809-12818 (2014).

[13] M. Mukaddam, E. Litwiller,. Pinnau, Gas sorption, diffusion, and permeation in nafion, Macromolecules, 49 (1), 280-286 (2015).

[14] Y.F. Fan, D. Tongren, C.J. Cornelius, The role of a metal ion within Nafion upon its physical and gas transport properties, European polymer journal, 50, 271-278 (2014).

[15] W. Xie, Y. Jiao, Z.L. Cai, H.Y. Liu, L.L. Gong, W. Lai, L.L. Shan, S.J. Luo, Highly selective benzimidazole-based polyimide/ionic polyimide membranes for pure-and mixed-gas CO_2/CH_4 separation, Separation and Purification Technology, 282, 120091 (2022).

[16] C.M. Zimmerman, W.J. Koros, Polypyrrolones for membrane gas separations. i. structural comparison of gas transport and sorption properties, Journal of Polymer Science Part B: Polymer Physics, 37 (12), 1235-1249 (1999).

[17] L. Wang, Y. Li, P. Zhang, X.F. Chen, P. Nian, Y.B. Wei, H.S. Lu, X.H. Gu, X.R. Wang, Thermally rearranged poly (benzoxazole-co-imide) composite membranes on α-Al_2O_3 support for helium extraction from natural gas, Journal of Membrane Science, 657, 120614 (2022).

[18] H.F. Guo, J. Wei, Y.L. Ma, Z.K. Qin, X.H. Ma, R. Selyanchyn, B.D. Wang, X.Z. He, B. Tang, L. Yang, L. Yao, W.J. Jiang, Y.F. Zhuang, D.G. Yin, X. Li, Z.D. Dai, Carbon molecular sieve membranes fabricated at low carbonization temperatures with novel polymeric acid porogen for light gas separation, Separation and Purification Technology, 317, 123883 (2023).

[19] R. Swaidan, B.S. Ghanem, E. Litwiller, I. Pinnau, Pure-and mixed-gas CO_2/CH_4 separation properties of PIM-1 and an amidoxime-functionalized PIM-1, Journal of membrane science, 457, 95-102 (2014).

[20] Y. Jiao, M.D. Liu, Q. Wu, P.J. Zheng, W. Xu, B.J. Ye, H.J. Zhang, R.L. Guo, S.J. Luo, Finely tuning the microporosity in phosphoric acid doped triptycene-containing polybenzimidazole membranes for highly permselective helium and hydrogen recovery, Journal of Membrane Science, 672, 121474 (2023).

[21] Z.Y. Zhang, Z. Yang, S.W. Li, L.B. Qian, X.L. Chen, G.N. Chen, G.P. Liu, W.Q. Jin, Hot solution strategy to prepare Zr-MOF/polyimide mixed matrix membranes for high-performance helium separation, Journal of Membrane Science, 729, 124137 (2025).

[22] J. Bai, L.Q. Xiao, H.Y. Liu, C. Wang, T.L. Han, L.L. Gong, S.J. Luo, H.W. Fan, L.L. Shan, High load mixed matrix membrane by metal-organic polyhedra for enhanced gas separation, Journal of Membrane Science, 733, 124322 (2025).

[23] A. Komal, L. Calderón-Rodríguez, O. Smirnova, E. Grossmann, A.B. Varghese, K.M.G. Alvarez, A. Schneemann, T. Hoyer, R. Wyrwa, Ideal Molecular Sieving with a Dense MOF for Helium Upgrading with Highly Diffusion Selective Mixed Matrix Membranes, Advanced Functional Materials, 2423999 (2024).

[24] X.H. Ma, K.H. Li, Z.Y. Zhu, H. Dong, J. Lv, Y.G. Wang, I. Pinnau, J.X. Li, B.W. Chen, Y. Han, High-performance polymer molecular sieve membranes prepared by direct fluorination for efficient helium enrichment, Journal of Materials Chemistry A, 9 (34), 18313-18322 (2021).

[25] M. Carta, R. Malpass-Evans, M. Croad, Y. Rogan, J.C. Jansen, P. Bernardo, F. Bazzarelli, N.B. Mckeown, An efficient polymer molecular sieve for membrane gas separations, Science, 339 (6117), 303-307 (2013).

[26] R. Williams, L.A. Burt, E. Esposito, J.C. Jansen, E. Tocci, C. Rizzuto, M. Lanč, M. Carta, N.B. Mckeown, A highly rigid and gas selective methanopentacene-based polymer of intrinsic microporosity derived from Tröger's base polymerization, Journal of Materials Chemistry A, 6 (14), 5661-5667 (2018).

[27] I. Rose, C.G. Bezzu, M. Carta, B. Comesaña-Gándara, E. Lasseuguette, M.C. Ferrari, P. Bernardo, G. Clarizia, A, Fuoco, J.C. Jansen, K.E. Hart, T.P. Liyana-Arachchi, C.M. Colina, N.B. McKeown, Polymer ultrapermeability from the inefficient packing of 2D chains, Nature Materials, 16 (9), 932-937 (2017).

[28] E.V. Perez, K.J. Balkus Jr, J.P. Ferraris, I.H. Musselman, Mixed-matrix membranes containing MOF-5 for gas separations, Journal of Membrane Science, 328 (1-2), 165-173 (2009).

[29] F. Weigelt, P. Georgopanos, S. Shishatskiy, V. Filiz, T. Brinkmann, V. Abetz, Development and characterization of defect-free Matrimid® mixed-matrix membranes containing activated carbon particles for gas separation, Polymers, 10 (1), 51 (2018).

[30] Y.F. Zhang, K.J. Balkus Jr, I.H. Musselman, J.P. Ferraris, Mixed-matrix membranes composed of Matrimid® and mesoporous ZSM-5 nanoparticles, Journal of Membrane Science, 325 (1), 28-39 (2008).

[31] M.J.C. Ordoñez, K.J. Balkus Jr, J.P. Ferraris, I.H. Musselman, Molecular sieving realized with ZIF-8/Matrimid® mixed-matrix membranes, Journal of Membrane Science, 361 (1-2), 28-37 (2010).

[32] A. Mirzaei, A.H. Navarchian, S. Tangestaninejad. Mixed matrix membranes on the basis of Matrimid and palladium-zeolitic imidazolate framework for hydrogen separation, Iranian Polymer Journal, 29, 479-491 (2020).

[33] X. Jiang, S.S. He, G. Han, J. Long, S.W. Li, C.H. Lau, S. Zhang, L. Shao, Aqueous one-step modulation for synthesizing monodispersed ZIF-8 nanocrystals for mixed-matrix membrane, ACS Applied Materials and Interfaces, 13 (9), 11296-11305 (2021).

[34] Y.F. Zhang, I.H. Musselman, J.P. Ferraris, K.J. Balkus, Jr, Gas permeability properties of mixed-matrix matrimid membranes containing a carbon aerogel: a material with both micropores and mesopores, Industrial & Engineering Chemistry Research, 47 (8), 2794-2802 (2008).

[35] Z.V. Singh, L.L. Tan, M.G. Cowan, Y.W. Yang, W. Zhang, D.L. Gin, R.D. Noble, Pillar [5] arene/Matrimid™ materials for high-performance methane purification membranes, Journal of Membrane Science, 539, 224-228 (2017).

[36] W. He, X.Z. Wang, J. Guan, Q.S. Liang, J. Ma, Y. Liu, W.W. Lim, C.W. Zhang, S.U. Hassan, H.J. Zhang, J.T. Liu, Membranes with Molecular Gatekeepers for Efficient CO_2 Capture and H_2 Purification, ACS Applied Materials & Interfaces 16 (16) 21222-21232, (2024).

[37] Y.B. Zhuang, J.G. Seong, Y.S. Do, W.H. Lee, M.J. Lee, M.D. Guiver, Y.M. Lee, High-strength, soluble polyimide membranes incorporating Tröger’s Base for gas separation, Journal of Membrane Science, 504, 55-65 (2016).

[38] J.J. Kong, J.J. Liu, P.Y. Jia, N. Qi, Z.Q. Chen, S. Xu, N.W. Li, Synergistic effect of thermal crosslinking and thermal rearrangement on free volume and gas separation properties of 6FDA based polyimide membranes studied by positron annihilation, Journal of Membrane Science, 645, 120163 (2022).

[39] X.F. Hu, W.H. Lee, J.Y. Bae, J.S. Kim, J.T. Jung, H.H. Wang, H.J. Park, Y.M. Lee, Thermally rearranged polybenzoxazole copolymers incorporating Tröger's base for high flux gas separation membranes, Journal of Membrane Science, 612, 118437 (2020).

[40] X.F. Hu, W.H. Lee, J.Y. Zhao, J.Y. Bae, J.S. Kim, Z. Wang, J.L. Yan, Y.B. Zhuang, Y.M. Lee, Tröger's Base (TB)-containing polyimide membranes derived from bio-based dianhydrides for gas separations, Journal of Membrane Science, 610, 118255 (2020).

[41] Z.G. Wang, D. Wang, F. Zhang, J. Jin, Troger’s base-based microporous polyimide membranes for high-performance gas separation, ACS Macro Letters, 3 (7), 597-601 (2014).

[42] B. Ghanem, N. Alaslai, X.H. Miao, I. Pinnau, Novel 6FDA-based polyimides derived from sterically hindered Tröger's base diamines: Synthesis and gas permeation properties, Polymer, 96, 13-19 (2016).

[43] S.S. Wu, J.C. Liang, Y.P. Shi, M.H. Huang, X.Y. Bi, Z.G. Wang, J. Jin, Design of interchain hydrogen bond in polyimide membrane for improved gas selectivity and membrane stability, Journal of Membrane Science, 618, 118659 (2021).

[44] Y.B. Zhuang, J.G. Seong, Y.S. Do, W.H. Lee, M.J. Lee, Z.L. Cui, A.E. Lozano, M.D. Guiver, Y.M. Lee, Soluble, microporous, Tröger's Base copolyimides with tunable membrane performance for gas separation, Chemical Communications, 52 (19), 3817-3820 (2016).

[45] R. Iwasa, T. Suizu, H. Yamaji, T. Yoshioka, K. Nagia, Gas separation in polyimide membranes with molecular sieve-like chemical/physical dual crosslink elements onto the top of surface, Journal of Membrane Science, 550, 80-90 (2018).

[46] M.J. Li, Z.B. Zheng, Z.G. Zhang, N.W. Li, S.W. Liu, Z.G. Chi, J.R. Xu, Y. Zhang, "All Polyimide" Mixed Matrix Membranes for High Performance Gas Separation, Polymers, 13 (8), 1329 (2021).

[47] S.N. Wijenayake, N.P. Panapitiya, S.H. Versteeg, C.N. Nguyen, S. Goel, K.J. Balkus, Jr, I.H. Musselman, J.P. Ferraris, Surface cross-linking of ZIF-8/polyimide mixed matrix membranes (MMMs) for gas separation, Industrial and Engineering Chemistry Research, 52 (21), 6991-7001 (2013).

[48] S.W. Zhu, Z.G. Wang, Y.S. Shi, W.K. Lai, Y.T. Zhang, J.Y. Jin, J. Jin, Benzyl-Induced Crosslinking of Polymer Membranes for Highly Selective CO_2/CH_4 Separation with Enhanced Stability, Macromolecules, 55 (15), 6890-6900 (2022).

[49] J.R. Wiegand, Z.P. Smith, Q. Liu, C.T. Patterson, B.D. Freeman, R.L. Guo, Synthesis and characterization of triptycene-based polyimides with tunable high fractional free volume for gas separation membranes, Journal of Materials Chemistry A, 2 (33), 13309-13320 (2014).

[50] S.H. Li, H.J. Jo, S.H. Han, C.H. Park, S. Kim, P.M. Budd, Y.M. Lee, Mechanically robust thermally rearranged (TR) polymer membranes with spirobisindane for gas separation, Journal of membrane science, 434, 137-147 (2013).

[51] J. Ahn, W.J. Chung, I. Pinnau, M.D. Guiver, Polysulfone/silica nanoparticle mixed-matrix membranes for gas separation, Journal of Membrane science, 314 (1-2), 123-133 (2008).

[52] Y.J. Wang, G. Yang, H.L. Guo, X.L. Meng, G.D. Kong, Z.X. Kang, R. Guillet-Nicolas, S. Mintova, Preparation of HKUST-1/PEI mixed-matrix membranes: Adsorption-diffusion coupling control of small gas molecules, Journal of Membrane Science, 643, 120070 (2022).

[53] A.K. Itta, H.H. Tseng, M.Y. Wey, Fabrication and characterization of PPO/PVP blend carbon molecular sieve membranes for H_2/N_2 and H_2/CH_4 separation. Journal of Membrane Science, 372 (1-2), 387-395 (2011).

[54] F.Y. Li, Y. Li, T.S. Chung, S. Kawi, Facilitated transport by hybrid POSS®-Matrimid®-Zn^{2+} nanocomposite membranes for the separation of natural gas, Journal of Membrane Science, 356 (1-2), 14-21 (2010).

[55] M.J.C. Ordoñez, K.J. Balkus Jr, J.P. Ferraris, I.H. Musselman, Molecular sieving realized with ZIF-8/Matrimid® mixed-matrix membranes, Journal of Membrane Science, 361 (1-2), 28-37 (2010).

[56] D.Q. Vu, W.J. Koros, S. J. Miller. Mixed matrix membranes using carbon molecular sieves: I. Preparation and experimental results, Journal of membrane science, 211 (2), 311-334 (2003).

[57] M. Waqas Anjum, F. Vermoortele, A.L. Khan, B. Bueken, D.E. De Vos, I.F.J. Vankelecom, Modulated UiO-66-based mixed-matrix membranes for CO_2 separation, ACS Applied Materials & Interfaces, 7 (45), 25193-25201 (2015).

[58] V. Martin-Gil, A. López, P. Hrabanek, R. Mallada, I.F.J. Vankelecom, V. Fila, Study of different titanosilicate (TS-1 and ETS-10) as fillers for Mixed Matrix Membranes for CO_2/CH_4 gas separation applications, Journal of Membrane Science, 523, 24-35 (2017).

[59] X.Y. Dong, Q. Liu, A.S. Huang. Highly permselective MIL-68 (Al)/matrimid mixed matrix membranes for CO_2/CH_4 separation, Journal of Applied Polymer Science, 133 (22), (2016).

[60] X.Q. Li, L. Ma, H.Y. Zhang, S.F. Wang, Z.Y. Jiang, R.L. Guo, H. Wu, X.Z. Cao, J. Yang, B.Y. Wang, Synergistic effect of combining carbon nanotubes and graphene oxide in mixed matrix membranes for efficient CO_2 separation, Journal of Membrane Science, 479, 1-10 (2015).

[61] M. Rahmani, A. Kazemi, F. Talebnia, Matrimid mixed matrix membranes for enhanced CO_2/CH_4 separation, Journal of Polymer Engineering, 36 (5), 499-511 (2016).

[62] M. Ahmadi, E. Taş, A. Kılıç, V. Kumbaracı, N. Talınlı, M.G. Ahunbay, Ş.B. Tantekin-Ersolmaz, Highly CO_2 selective microporous metal-imidazolate framework-based mixed matrix membranes, ACS Applied Materials & Interfaces, 9 (41), 35936-35946 (2017).

[63] M. Peydayesh, S. Asarehpour, T. Mohammadi, O. Bakhtiari, Preparation and characterization of SAPO-34-Matrimid® 5218 mixed matrix membranes for CO_2/CH_4 separation, Chemical Engineering Research and Design, 91 (7), 1335-1342 (2013).

[64] S. Kanehashi, G.Q. Chen, C.A. Scholes, B. Ozcelik, C. Hua, L. Ciddor, P.D. Southon, D.M. D'Alessandro, S.E. Kentish, Enhancing gas permeability in mixed matrix membranes through tuning the nanoparticle properties, Journal of Membrane Science, 482, 49-55 (2015).

[65] S. Shahid, K. Nijmeijer, Performance and plasticization behavior of polymer-MOF membranes for gas separation at elevated pressures, Journal of membrane science, 470, 166-177 (2014).

[66] A.E. Amooghin, H. Sanaeepur, M. Omidkhah, A. Kargari, "Ship-in-a-bottle", a new synthesis strategy for preparing novel hybrid host-guest nanocomposites for highly selective membrane gas separation, Journal of Materials Chemistry A, 6 (4), 1751-1771 (2018).

[67] M.W. Anjum, F. De Clippel, J. Didden, A.L. Khan, S. Couck, G.V. Baron, J.F.M. Denayer, B.F. Sels, I.F.J. Vankelcome, Polyimide mixed matrix membranes for CO_2 separations using carbon-silica nanocomposite fillers, Journal of Membrane Science, 495, 121-129 (2015).

[68] F. Moghadam, M.R. Omidkhah, E. Vasheghani-Farahani, M.Z. Pedram, F. Dorosti, The effect of TiO_2 nanoparticles on gas transport properties of Matrimid 5218-based mixed matrix membranes, Separation and Purification Technology, 77 (1), 128-136 (2011).

[69] J. Ahmad, M.B. Hägg, Development of matrimid/zeolite 4A mixed matrix membranes using low boiling point solvent, Separation and Purification Technology, 115, 190-197 (2013).

[70] C.I. Chaidou, G. Pantoleontos, D.E. Koutsonikolas, S.P. Kaldis, G.P. Sakellaropoulos, Gas separation properties of polyimide-zeolite mixed matrix membranes, Separation Science and Technology, 47 (7), 950-962 (2012).

[71] Q.L. Song, S.K. Nataraj, M.V. Roussenova, J.C. Tan, D.J. Hughes, W. Li, P. Bourgoin, M.A. Alam, A.K. Cheetham, S.A. Al-Muhtaseb, E. Sivaniah, Zeolitic imidazolate framework (ZIF-8) based polymer nanocomposite membranes for gas separation, Energy & Environmental Science, 5 (8), 8359-8369 (2012).

[72] H. Asghar, A. Ilyas, Z. Tahir, X.F. Li, A.L. Khan, Fluorinated and sulfonated poly (ether ether ketone) and Matrimid blend membranes for CO_2 separation, Separation and Purification Technology, 203, 233-241 (2018).

[73] S.R. Venna, M. Lartey, T. Li, A. Spore, S. Kumar, H.B. Nulwala, D.R. Luebke, N.L. Rosi, E. Albenze, Fabrication of MMMs with improved gas separation properties using externally-functionalized MOF particles, Journal of Materials Chemistry A, 3 (9), 5014-5022 (2015).

[74] S.S. Hosseini, T.S. Chung, Carbon membranes from blends of PBI and polyimides for N_2/CH_4 and CO_2/CH_4 separation and hydrogen purification, Journal of Membrane Science, 328 (1-2), 174-185 (2009).

[75] J.Y. Liu, C.R.P. Fulong, L.Q. Hu, L. Huang, G.Y. Zhang, T.R. Cook, H.Q. Lin, Interpenetrating networks of mixed matrix materials comprising metal-organic polyhedra for membrane CO_2 capture, Journal of Membrane Science, 606, 118122 (2020).

[76] D. Husken, T. Visser, M. Wessling, R.J. Gaymans, CO_2 permeation properties of poly (ethylene oxide)-based segmented block copolymers, Journal of membrane science, 346 (1), 194-201 (2010).

[77] N.C. Su, Z.P. Smith, B.D. Freeman, J.J. Urban, Size-dependent permeability deviations from Maxwell's model in hybrid cross-linked poly (ethylene glycol)/silica nanoparticle membranes, Chemistry of Materials, 27 (7), 2421-2429 (2015).

[78] L.Q. Hu, J.Y. Liu, L.X. Zhu, X.D. Hou, L. Huang, H.Q. Lin, J. Cheng, Highly permeable mixed matrix materials comprising ZIF-8 nanoparticles in rubbery amorphous poly (ethylene oxide) for CO_2 capture, Separation and Purification Technology, 205, 58-65 (2018).

[79] X.G. Li, I. Kresse, Z.K. Xu, J. Springer, Effect of temperature and pressure on gas transport in ethyl cellulose membrane, Polymer, 42 (16), 6801-6810 (2001).

[80] G. Perego, A. Roggero, R. Sisto, C. Valentini, Membranes for gas separation based on silylated polyphenylene oxide, Journal of membrane science, 55 (3), 325-331 (1991).

[81] W.J. Chen, C.R. Martin. Gas-transport properties of sulfonated polystyrenes, Journal of membrane science, 95 (1), 51-61 (1994).

[82] M.W. Hellums, W.J. Koros, G.R. Husk, D.R. Paul, Fluorinated polycarbonates for gas separation applications, Journal of Membrane Science, 46 (1), 93-112 (1989).

[83] J.S. McHattie, W.J. Koros, D.R. Paul, Gas transport properties of polysulphones: 1. Role of symmetry of methyl group placement on bisphenol rings, Polymer, 32 (5), 840-850 (1991).

[84] J.T. Li, K. Nagai, T. Nakagawa, S.C. Wang, Preparation of polyethyleneglycol (PEG) and cellulose acetate (CA) blend membranes and their gas permeabilities, Journal of applied polymer science, 58 (9), 1455-1463 (1995).

[85] L. Hao, P. Li, T.S. Chung, PIM-1 as an organic filler to enhance the gas separation performance of Ultem polyetherimide, Journal of membrane science, 453, 614-623 (2014).

[86] S.Y. Hwang, W.S. Chi, S.J. Lee, S.H. Im, J.H. Kim, J. Kim, Hollow ZIF-8 nanoparticles improve the permeability of mixed matrix membranes for CO_2/CH_4 gas separation, Journal of Membrane Science, 480, 11-19 (2015).

[87] C.J. Duan, X.M. Jie, D.D. Liu, Y.M. Cao, Q. Yuan, Post-treatment effect on gas separation property of mixed matrix membranes containing metal organic frameworks, Journal of membrane science, 466, 92-102 (2014).

[88] J.L. Wang, Y.H. Ding, M. He, X.D. Ding, X. Liu, W.Q. Shi, Direct Preparation of Ultrathin Polymer Membranes on Porous Substrates for the Separation of Helium From Methane, Small, 21 (4), 2406440 (2025).

[89] T.A. Centeno, A.B. Fuertes, Carbon molecular sieve gas separation membranes based on poly (vinylidene chloride-co-vinyl chloride), Carbon, 38 (7), 1067-1073 (2000).

[90] W. Ogieglo, T. Puspasari, M.K. Hota, N. Wehbe, H.N. Alshareef, I. Pinnau, Nanohybrid thin-film composite carbon molecular sieve membranes, Materials Today Nano, 9, 100065 (2020).

[91] C. Feng, H.F. Guo, M. Deng, J. Wei, Y.L. Ma, Z.K. Qin, X.H. Ma, J. Liu, L.Y. Deng, W.J. Jiang, L. Yang, L. Yao, Z.D. Dai, Thin-film-composite carbon molecular sieve membranes for efficient helium and hydrogen separation, Industrial & Engineering Chemistry Research, 63 (1), 594-606 (2023).

[92] S. Husain, W.J. Koros, Mixed matrix hollow fiber membranes made with modified HSSZ-13 zeolite in polyetherimide polymer matrix for gas separation, Journal of Membrane Science, 288 (1-2), 195-207 (2007).

[93] P.K. Gantzel, U. Merten, Gas separations with high-flux cellulose acetate membranes, Industrial & Engineering Chemistry Process Design and Development, 9 (2), 331-332 (1970).

[94] S.K. Choi, M.M.B. Sultan, A.A. Alsuwailem, S.M. Zuabi, Preparation and characterization of multilayer thin-film composite hollow fiber membranes for helium extraction from its mixtures, Separation and Purification Technology, 222, 152-161 (2019).

[95] G. Dibrov, M. Ivanov, M. Semyashkin, M. Semyashkin, V. Sudin, G. Kagramanov, High-pressure aging of asymmetric Torlon® hollow fibers for helium separation from natural gas, Fibers, 6 (4), 83 (2018).

[96] E.K. Chatzidaki, E.P. Favvas, S.K. Papageorgiou, N.K. Kanellopoulos, N.V. Theophilou, New polyimide-polyaniline hollow fibers: synthesis, characterization and behavior in gas separation, European Polymer Journal, 43 (12), 5010-5016 (2007).

[97] W. Ogieglo, T. Puspasari, X.H. Ma, I. Pinnau, Sub-100 nm carbon molecular sieve membranes from a polymer of intrinsic microporosity precursor: Physical aging and near-equilibrium gas separation properties, Journal of Membrane Science, 597, 117752 (2020).

[98] C. Zhang, W.J. Koros, Ultraselective carbon molecular sieve membranes with tailored synergistic sorption selective properties, Advanced Materials, 29 (33), 1701631 (2017).

[99] S.B. Carruthers, G.L. Ramos, W.J. Koros, Morphology of integral-skin layers in hollow-fiber gas-separation membranes, Journal of applied polymer science, 90 (2), 399-411 (2003).

[100] Q. Li, Z.H. Guo, S.N. Geng, M.Y. Wen, J.H. Wang, L. Yu, Precision tuning of micropore flexibility in polysilsesquioxane membranes for enhanced helium extraction, Chemical Engineering Journal, 168294 (2025).

[101] S. Takamizawa, Y. Takasaki, R. Miyake, Single-crystal membrane for anisotropic and efficient gas permeation, Journal of the American Chemical Society, 132 (9), 2862-2863 (2010).

[102] Y. Yoo, V. Varela-Guerrero, H.K. Jeong, Isoreticular metal-organic frameworks and their membranes with enhanced crack resistance and moisture stability by surfactant-assisted drying, Langmuir, 27 (6), 2652-2657 (2011).

[103] Ranjan R, Tsapatsis M. Microporous metal organic framework membrane on porous support using the seeded growth method, Chemistry of Materials, 21 (20), 4920-4924 (2009).

[104] Z.J. Zhao, L. Ding, A. Mundstock, O. Stölting, S. Polarz, H.H. Wang, A. Feldhoff, Preparation of ZIF-62 polycrystalline and glass membranes for helium separation, Journal of Membrane Science, 700, 122677 (2024).

[105] S. Denning, J. Lucero, C.A. Koh, M.A. Carreon, Chabazite zeolite SAPO-34 membranes for He/CH_4 separation, ACS Materials Letters, 1 (6), 655-659 (2019).

[106] D.F. Liu, X.L. Ma, H.X. Xi, Y.S. Lin, Gas transport properties and propylene/propane separation characteristics of ZIF-8 membranes, Journal of membrane science,451, 85-93 (2014).

[107] J. Bai, L.Q. Xiao, X.P. Zhang, H.Y. Liu, C. Wang, L.L. Gong, S.J. Luo, Y.H. Zhu, L.L. Shan, H.W. Fan, S.J. Zhang, Metallocene-anchor inducing oriented MOF membrane for helium separation, Nature Communications, 16 (1), 9451 (2025).

[108] M.F. Fang, Y. Okamoto, Y. Koike, Z.J. He, T.C. Merkel, Gas separation membranes prepared with copolymers of perfluoro (2-methylene-4, 5-dimethyl-1, 3-dioxlane) and chlorotrifluoroethylene, Journal of Fluorine Chemistry, 188, 18-22 (2016).

[109] M.F. Fang, Z.J. He, T.C. Merkel, Y. Okamoto, High-performance perfluorodioxolane copolymer membranes for gas separation with tailored selectivity enhancement, Journal of Materials Chemistry A, 6 (2), 652-658 (2018).

[110] X.R. Wang, M.X. Shan, X.L. Liu, M. Wang, C.M. Doherty, D. Osadchii, F. Kaptrijn, High-performance polybenzimidazole membranes for helium extraction from natural gas, ACS Applied Materials & Interfaces, 11 (22), 20098-20103 (2019).

[111] W.L. Zhou, M. Yoshino, H. Kita, K. Okamoto, Preparation and gas permeation properties of carbon molecular sieve membranes based on sulfonated phenolic resin, Journal of Membrane Science, 217 (1-2), 55-67 (2003).
